# Effectiveness of remote gamification pulmonary rehabilitation intervention based on the health action process approach theory in older adults with chronic obstructive pulmonary disease: a pilot randomized controlled trial

**DOI:** 10.3389/fmed.2025.1576256

**Published:** 2025-06-19

**Authors:** Yuyu Jiang, Manyao Sun, Baiyila Nuerdawulieti, Xueying Huang, Yi Hou, Jiang Nan, Shiya Cui, Xun Nan

**Affiliations:** ^1^Research Office of Chronic Disease Management and Rehabilitation, Department of Nursing, Wuxi School of Medicine, Jiangnan University, Wuxi, China; ^2^School of Medicine Xinjiang College of Science & Technology, Korla, China

**Keywords:** chronic obstructive pulmonary disease, randomized controlled trial, gamification, behavior change, telemedicine

## Abstract

**Background:**

Remote pulmonary rehabilitation (PR) is widely used in the management of chronic obstructive pulmonary disease (COPD), but there is a problem of adherence. Health Action Process Theory (HAPA) is an effective behavior change theory, and combining it with gamification is expected to further improve PR adherence. This study explored the improvement effect of remote gamification PR intervention based on HAPA theory on rehabilitation adherence, clinical symptoms, quality of life and psychological outcome indicators of COPD patients, and compared it with self-efficacy theory and HAPA theory based remote PR.

**Methods:**

159 COPD patients were randomly divided into three groups: PR group, HAPA-PR group, and HAPA-gamification-PR (HAPA-Ga-PR) group. All three groups received 12 weeks of intervention and 12 weeks of follow-up. The primary outcome indicators were quality of life of patients and PR adherence. Secondary outcome measures were dyspnea symptoms, exercise self-efficacy, exercise motivation and positive affect.

**Results:**

A total of 147 patients completed the experiment. At the 12th week of intervention, there were statistically significant differences in PR adherence (*p* = 0.015), exercise self-efficacy (*p* = 0.039), exercise motivation (*p* = 0.008) and positive affect (*p* = 0.004) between the PR group and the HAPA-Ga-PR group. There were statistically significant differences in exercise motivation (*p* = 0.044) and positive affect (*p* = 0.046) between HAPA-PR group and HAPA-Ga-PR group. At week 24, there were statistically significant differences in quality of life (*p* = 0.039), PR adherence (*p* = 0.001), exercise motivation (*p* = 0.027) and positive affect (*p* = 0.015) between the PR group and the HAPA-Ga-PR group. Compared to baseline, at week 24, only the HAPA-Ga-PR group showed statistically significant improvement in exercise self-efficacy (*p* = 0.013) in COPD patients.

**Conclusion:**

Remote gamification PR intervention based on HAPA theory shows significant advantages in improving PR adherence, quality of life and psychological outcome of COPD patients, providing a new way for chronic disease management and personalized digital health services. This model can be extended to more chronic disease rehabilitation scenarios in the future.

**Clinical Trial Registration:**

Chinese Clinical Trial Registry (ChiCTR): ChiCTR1900028563; https://www.chictr.org.cn/index.html.

## 1 Introduction

Chronic obstructive pulmonary disease (COPD) is a common chronic respiratory disease with a global prevalence of about 10.3%. It ranks as the third leading cause of death worldwide (1). The incidence of COPD increases dramatically with age and is highest in people over 60 years of age (2). Pulmonary rehabilitation (PR) is one of the standard and most cost-effective treatments for COPD (3). Due to challenges such as distance, transportation, the limited number of rehabilitation programs, and inadequate resources, remote PR has been gradually promoted (4). In recent years, with the popularity of smart devices, more and more older adults in China have begun to use the Internet. By 2023, the number of Internet users aged 60 and above has increased to 140 million (5). In addition, due to the COVID-19 pandemic, the Chinese government has taken several measures to help the older adults overcome difficulties in using smart technology (6). This trend has laid a foundation for the development of remote PR. Although remote PR programs have achieved some success, the low adherence of COPD patients remains a significant concern (7). A cluster RCT reported that only 46% of patients completed over 70% of their rehabilitation sessions (8). The standard course of remote PR spans several months, but once exercise training stops, the benefits achieved during rehabilitation tend to diminish within months after the program ends (9). Therefore, improving adherence to PR in COPD patients under remote settings is crucial.

PR adherence belongs to the category of health behaviors and can be achieved through behavioral interventions. Among emerging tele-health behavior interventions, the Health Action Process Approach (HAPA) has been proven effective in promoting health behavior change and maintenance (10, 11). This theory posits that behavior change is a continuous process involving initiation, maintenance, and recovery after interruption, consistent with behavior changes in PR (12). HAPA explains health behavior changes through psychological factors such as risk awareness, behavioral beliefs, behavioral intentions, and self-efficacy, emphasizing the generation, maintenance, and recovery of behaviors (12). Self-efficacy plays a critical role throughout the behavior process, with intervention strategies including direct experience, indirect experience, verbal persuasion, and the management of affectal and physiological states (13). However, among online interventions based on the HAPA theory, most existing studies are conducted in the form of manuals, SMS, phone calls and emails, lacking visual feedback. For example, interventions aimed at direct experience help users experience success through progressively more difficult tasks and activities, but lack timely visual feedback to let users perceive their progress (14). Therefore, in remote settings, it is essential to enhance interactivity, personalized feedback, and immediate visual feedback to achieve more effective self-efficacy interventions.

In the remote behavior intervention, gamification is widely used and characterized by interactivity, personalized feedback, and instant visual feedback (15, 16). Gamification refers to the application of game design elements and mechanics in a non-game environment. Common gamification elements are divided into three main categories: achievement-oriented (e.g., badges, points, leaderboards), which promote immediate feedback and a sense of accomplishment, and motivate continuous engagement; immersive-oriented (e.g., narrative, avatar) to enhance user experience and immersion; social-oriented (e.g., cooperation, social support) to enhance user engagement and motivation (17). In the self-management of patients with various chronic diseases such as osteoarthritis, diabetes, and coronary heart disease, the role of gamification has been proven to improve patient adherence and health outcome indicators, indicating a broad application prospect (18–20). However, most researches in the field of health lacked a theoretical basis for gamification interventions, and some scholars advocated for the need for more theory-driven gamification research (21). Therefore, it is worth considering the integration of gamification with HAPA to innovate self-efficacy intervention strategies within the HAPA theory through the incorporation of gamification elements.

In addition, previous studies have shown that both HAPA theory and gamification can influence key psychological indicators, such as positive affect and motivation. HAPA theory enhances individual health behavior by improving self-efficacy, action intention, and plan realization, a process closely linked to the enhancement of positive affect, which helps maintain the momentum for behavior change (12). Similarly, gamification can stimulate intrinsic motivation through challenging tasks and feedback systems, while rewards may drive extrinsic motivation (22), and all of these elements can enhance user engagement and affect experience (23). Therefore, this study aims to explore whether remote gamification PR intervention based on HAPA theory can effectively promote positive affect and motivation in patients with COPD, ultimately improving PR adherence and quality of life.

The objective of this study is to develop and evaluate the remote PR intervention based on gamification and HAPA theory for older adults with COPD, and to explore the mechanism of its effects in terms of self-efficacy, positive emotion and motivation.

## 2 Materials and methods

This study was a 24-week three-arm pilot randomized controlled trial, divided into standard PR group, HAPA-based PR group (HAPA-PR) and HAPA-based gamification-combined PR group (HAPA-Ga-PR). Each group included a 12-week intervention period and a 12-week follow-up period. Remote intervention relies on WeChat and Pulmonary Internet Explorer Rehabilitation (PeR, a free social media WeChat public account) previously built by our research group (24). According to the results of the sequence, the research assistant randomly divided the patients into three groups: PR group, HAPA-PR group, and HAPA-Ga-PR group. Data collection also followed the principle of blindness, carried out by research assistants who did not know about patient groupings. The interventions in the three groups were conducted simultaneously to eliminate the influence of weather and season on COPD patients. This study was approved by the Medical Ethics Committee of Jiangnan University (JNU20220310IRB17), and has been registered in the Chinese Clinical Trial Registry (ChiCTR 1,900,028,563). The principle of informed consent was followed in this study. See Supplementary material 1 for checklist.

### 2.1 Recruitment and participants

Patient recruitment was conducted between August 7, 2023 and May 11, 2024, using leaflets and posters to recruit patients in the outpatient and inpatient departments of six hospitals in Wuxi City. The eligibility criteria were: (1) age 65 or above; (2) according to the COPD diagnosis and management guidelines (25), the patient was evaluated by clinicians and rehabilitation therapists as being in stable COPD and meeting the criteria of forced expiratory volume in 1 s to forced vital capacity ratio (FEV1/FVC) less than 0.70 and FEV1 less than 80% predictive value; (3) the ability to use or share smart phones. Exclusion criteria include: (1) auditory, visual or verbal inability to communicate; (2) suffering from a mental disorder, cognitive impairment or severe physical impairment; (3) suffering from heart disease or arrhythmia requiring medical treatment; (4) have poorly controlled high blood pressure; (5) a history of syncope after exercise.

### 2.2 Sample size

The main evaluation indicator assessed in this study was quality of life, measured using the COPD Assessment Test (CAT). Based on the effect size (Cohen’s d = 0.4), *α* = 0.05 (double tail), *β* = 0.2 (80% efficacy) and 15% drop rate for PR in previous studies, 174 people were calculated for each group in the formal trial (24, 26). However, the actual effect size of remote PR may be larger (Cohen’s d = 0.7) (27), and the sample size requirement can be reduced to 30% of the original value, which means 53 participants per group to still meet the statistical efficacy. In addition, combined with the pilot study, it is recommended that the common sample size is 10–50 people per group (28). In this study, the pilot sample size is set at a minimum of 53 participants per group, resulting in a total sample size of 159 participants.

### 2.3 Development of remote gamification PR component based on HAPA theory

**Figure 1:**
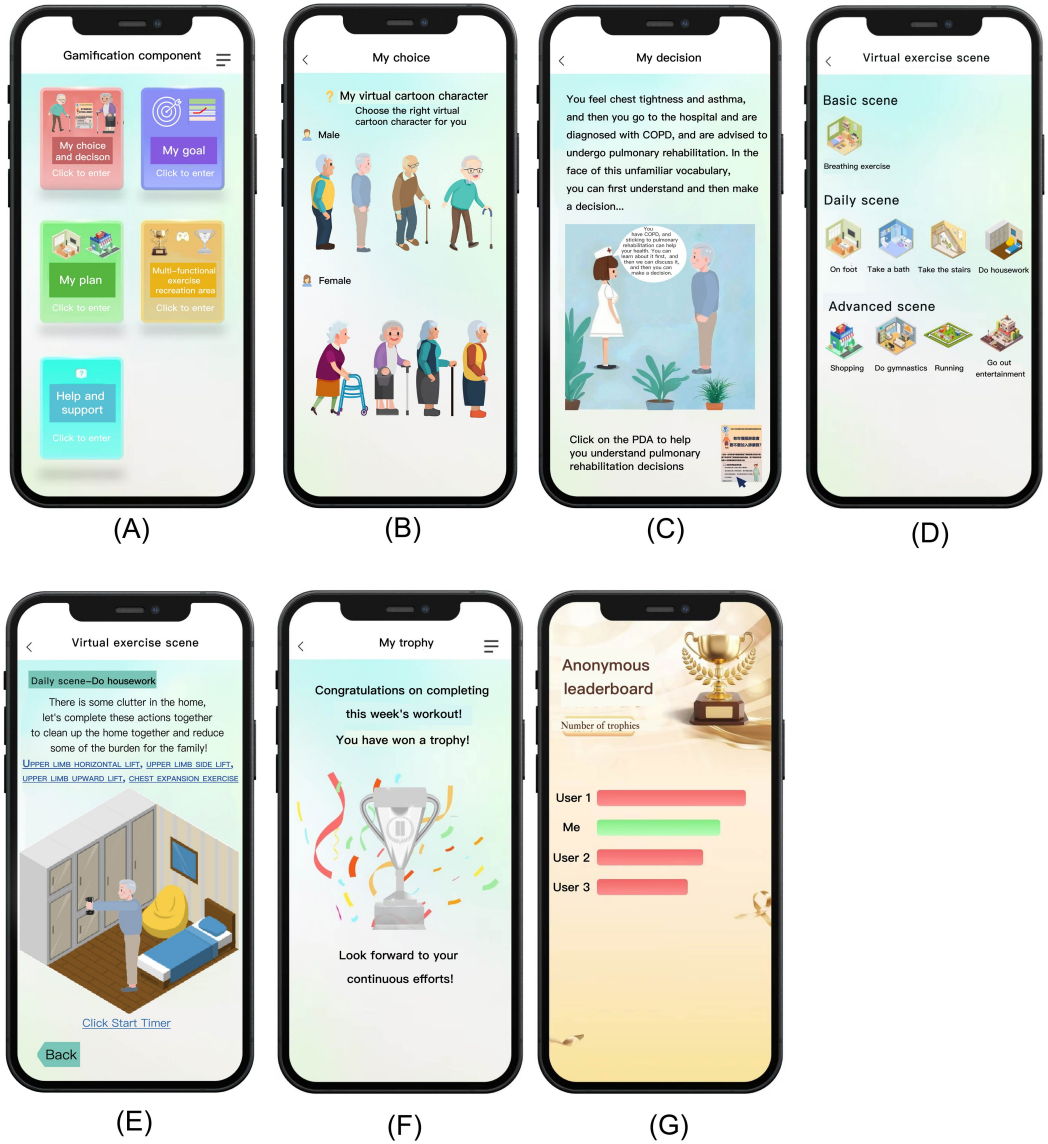
Interface display of gamification pulmonary rehabilitation component based on HAPA theory.

This study is based on the existing PeR, which includes a basic PR intervention program characterized by self-efficacy, and the intervention effect has been proven (24). There is an “energy zone” component in the program, which can be expanded to achieve different interventions with different characteristics. In this study, a patient decision aid (PDA) and a gamification component were added to the “energy zone” component. The effectiveness of PDA has been proven (29). Gamification component was developed using an agile development model. Gamification component was based on the results of literature research and are designed and developed by multidisciplinary teams through focus group discussions. After the completion of the initial development of gamification components, alpha testing was conducted first, expert opinions were collected, and the rationality of functions and designs was evaluated through two rounds of expert correspondence, and modifications were made. This was followed by the beta testing phase, where, in combination with a hybrid research approach, the components were systematically evaluated for usability, with a focus on effectiveness, efficiency and satisfaction indicators. Its effectiveness was tested by task completion rate. Efficiency was tested by task completion time and customer effort score. Satisfaction was measured using post-study system usability questionnaire and net promoter score. The usability test showed that the task completion rate of gamification components reached 100%, which indicated the complete feasibility of functional design. The average of customer effort score was 3.05 and the average of net promoter score was 4.50, reflecting high operational efficiency and user satisfaction. The specific content of usability testing and the theoretical mapping of the scheme design are shown in Supplementary material 2. The final interface display is shown in Figure 1.

### 2.4 Intervention

#### 2.4.1 PR group

A 12-week remote PR program was carried out, which was jointly implemented by a multidisciplinary team (rehabilitation therapists, doctors and nurses), including providing patients with comprehensive assessment and exercise tests, formulating exercise prescriptions, issuing rehabilitation diaries, guiding patients to learn relevant knowledge, and discussing obstacles and coping strategies that may affect the rehabilitation plan (24). Patients uploaded training photos or videos to record their progress through the PeR system and earned points redeemable in kind. Patients interacted with nurses by commenting or liking (24). Nurses checked rehabilitation of patients every 4 weeks via WeChat video. The patients recorded the number of movement steps through the sensor of the mobile phone. Before recording the number of movement steps, the patients needed to sit still for 10 min or more to ensure the accuracy of the recorded data.

#### 2.4.2 HAPA-PR group

Based on the PR group, HAPA-PR group used the PDA component (29). Combined with HAPA theory, the intervention was divided into three stages: (i) Behavioral intention stage: patients received PDA training and applied PDA to make decisions, and clarified behavioral beliefs. Nurses and patients jointly developed exercise rehabilitation goals in line with their own values (29); (ii) Planning stage: Jointly develop personalized plans according to patients’ conditions and preferences to enhance patients’ self-efficacy (29); (iii) Implementation stage: Patients were trained at home and reported their training status in writing every week. The patient’s exercise situation was summarized by the nurses’ feedback every 4 weeks to improve the self-efficacy of behavior maintenance and recovery.

#### 2.4.3 HAPA-Ga-PR group

Based on the HAPA-PR group, this group added “gamification components” in the “energy zone” to achieve gamification intervention, as shown in Figure 1A. To ensure that the patients could operate the component, at the first intervention, the patient watched and learned a pre-recorded video of the interface operation, which was approximately 120 s long, recorded by a multidisciplinary team. The intervention was divided into three stages: (i) Behavioral intention stage: During the interface operation, the patient first entered the “My choice and decision” module, as shown in Figures 1B,C. In this module, patients could select the most suitable virtual cartoon characters for themselves. Then, the virtual cartoon characters of the patients were used to share decision-making with healthcare providers to decide whether to carry out PR. The patients were asked to click the PDA link to assist the patients in PR decision-making. Through virtual cartoon characters and scenes, patients’ sense of immersion was enhanced, and patients’ risk awareness and behavioral beliefs were further enhanced. (ii) Planning stage: Patients entered the “My Plan” with virtual cartoon characters, and could choose virtual exercise scenes according to personal preferences, which were divided into basic scenes, daily scenes and advanced scenes [9 scenes in total, based on the St. George’s Breathing questionnaire (30)], and enhanced patients’ self-efficacy by enhancing immersion, as shown in Figure 1D. (iii) Implementation stage: Patient could enter the “Multi-functional exercise recreation area,” including “Virtual exercise scene,” “My Trophy,” “Anonymous Leaderboard” and “Rehabilitation Effect Perception Visualization” as shown in Figures 1E–G; Supplementary material 3. In the “Virtual exercise scene,” the system appeared the corresponding exercise animation scene according to the rehabilitation exercise plan formulated by the patients, and the patients could perform PR exercise according to the action video guidance of their virtual cartoon character on the interface, and recorded the time. In the “My Trophy” module, based on the patients’ weekly self-reported adherence results, the system gave the patient a gold trophy when the weekly adherence was 75% or above, and a silver trophy when the adherence was lower than 75%. If the exercise for three consecutive weeks was 75% or above, the system would give the “Persistence Talent Award.” In the “Anonymous Leaderboard,” patients could anonymously check the number of days that others had performed rehabilitation and the number of trophies they had won. When the patients had no rehabilitation record for more than 2 weeks, the system reminded patients that they had been surpassed in the leaderboard because they had not been exercising consistently. In the “Rehabilitation Effect Perception Visualization,” patients could perceive the rehabilitation effect by watching the current animated video of dyspnea of themselves virtual cartoon characters. These animated videos corresponded to the 5 degrees of the modified Medical Research Council (mMRC) scale (31), which effectively reflected the patients’ dyspnea conditions. Every 4 weeks, according to the degree of dyspnea by patients self-assessment, the system displayed the video of dyspnea. The above gamification elements and visual display of rehabilitation effects could improve patients’ self-efficacy in behavior maintenance and recovery.

### 2.5 Evaluation indicators

#### 2.5.1 Main evaluation indicators

The main evaluation indicators of this study were patients’ adherence with PR exercise and quality of life. In the process of PR, traditional pulmonary function indicators (such as FEV1/FVC) usually do not change significantly and may not fully reflect the patient’s quality of life and daily function status. In guideline, quality of life assessment tools were recommended to evaluate the effectiveness of patients as PR (3). Quality of life was assessed using the COPD Assessment Test (CAT) scale, which includes eight items related to cough, phlegm, chest tightness, limited activity at home, and high energy levels. CAT is mainly used to assess the impact of COPD on the health status of patients, and has a strong correlation with the quality of life of patients with COPD (32). Therefore, CAT is widely used in clinical practice as an effective tool to assess quality of life in patients with COPD. Each item is scored on a scale of 0 to 5 on a 0–40 scale, with higher scores indicating poorer health for the patient. PR adherence is collected through patient self-reports, which is a simple and commonly used method. Adherence is classified into high adherence and low adherence according to whether the proportion of actually completed rehabilitation exercises and plans reaches 75% (33).

#### 2.5.2 Secondary evaluation indicators

Exercise self-efficacy was assessed by the Exercise Self-Regulatory Efficacy Scale (Ex-SRES), a 16-item scale designed to measure whether patients were able to stick to exercise in the face of different difficulties. These challenges involve factors such as weather, physical condition, time, social support, oxygen, fatigue and mood. Each entry was scored on a 0–10 scale, with a higher score indicating greater patient confidence in exercise. Ex-SRES scale has a single factor structure and good internal consistency, with Cronbach *α* coefficient of 0.917 (34). The assessment of dyspnea was carried out through the mMRC dyspnea scale, which included five dimensions to measure the degree of dyspnea perceived by individuals. The higher the score, the more obvious the dyspnea perceived by patients (31). To evaluate patients’ positive affect, the Positive Affect subscale of the Positive and Negative Affect Schedule (PANAS) was used, demonstrating a Cronbach’s α coefficient of 0.85 and a test–retest reliability of 0.47. This scale consists of 10 positive affect descriptors as measurement indicators (35). Additionally, the Behavioral Regulation in Exercise Questionnaire-2 (BREQ-2) was employed to assess patients’ exercise motivation, consisting of 19 items. The dimensions included are Amotivation, External Regulation, Introjected Regulation, Identified Regulation, and Intrinsic Regulation. The patient’s relative autonomy score was calculated using a weighted approach based on the relative autonomy index, representing the degree of the patient’s exercise motivation (36).

### 2.6 Data collection

Baseline data, including patient demographics and evaluation indicators, were collected prior to the intervention. Adherence to PR was assessed at the end of the first week to establish baseline adherence levels. Indicators were further evaluated at the end of week 12 and week 24 to assess the intervention’s impact over time.

### 2.7 Data analysis

SPSS Statistics 27.0 (IBM) was used to analyze the data, and the statistical significance was *p* less than 0.05. Demographic and baseline data was described using descriptive analysis, Chi-square tests, Fisher tests, one-way analysis of variance (ANOVA), and Kruskal-Wallis H tests. Repeated measure ANOVA and generalized estimation equations were used to study the outcome changes at different intervention times (0, 12, 24 weeks). The baseline value of rehabilitation adherence was taken from the first week of intervention. Repeated measure ANOVA was used to compare the difference of outcome indicators in different intervention periods (Mann–Whitney U Test was used to compare the mMRC score; chi-square test was used to compare the adherence). Differences in change from baseline were compared between groups by McNemar test, estimate the marginal mean, or Wilcoxon rank sum test.

## 3 Results

### 3.1 Recruitment and research processes

**Figure 2:**
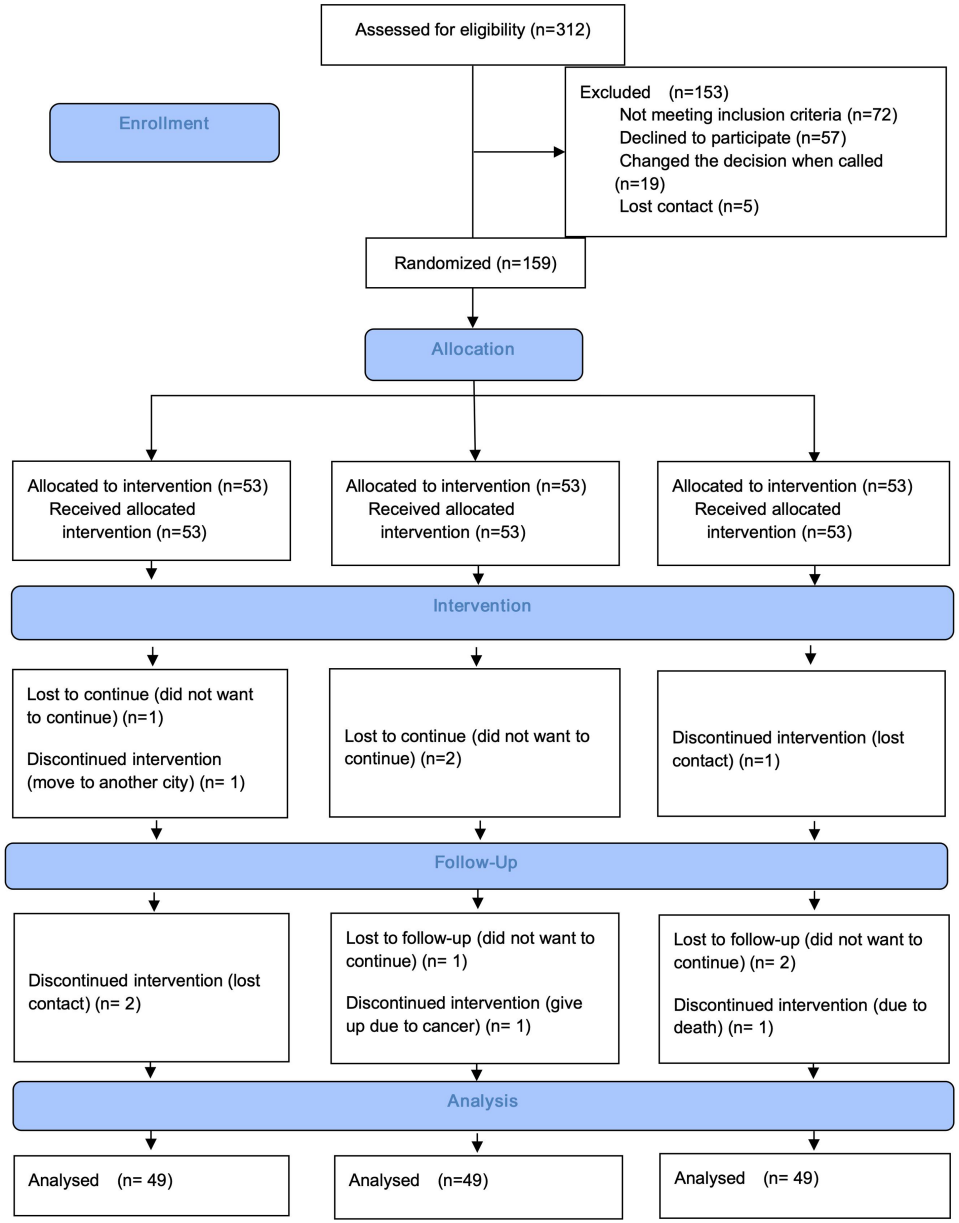
Consolidated standards of reporting trials flowchart.

The CONSORT flow chart of this study (Figure 2) shows the patient recruitment process, intervention implementation, and loss of follow-up. During the initial screening phase, a total of 312 patients were eligible for consideration, of which 184 (58.97% response rate) met the inclusion criteria and agreed to participate in the PR program. However, before the intervention began, 19 patients changed their intention to participate, and another 5 patients lost contact. Ultimately, 159 patients (50.96% response rate) were randomly assigned to one of three groups. During the intervention period, 4 patients (7.54%) in each group dropped out of the study.

### 3.2 Basic demographic characteristics and measurement

There were no significant differences in socio-demographic characteristics and baseline measurements among the PR group, HAPA-PR group, and HAPA-Ga-PR group (*p* > 0.05). Details is shown in Table 1.

**Table 1:** Baseline characteristics and measurements of participants.

Measurements	x̅±s / M (P_25_, P_75_) / n%				
	PR (*n* = 49)	HAPA-PR (*n* = 49)	HAPA-Ga-PR (*n* = 49)	*F/χ^2^*	*p*
Age					
	74.71 ± 4.71	75.73 ± 5.32	75.44 ± 4.48	0.576	0.563^a^
Sex					
					0.941^b^
Male	43 (87.76)	44 (89.80)	45 (91.80)		
Female	6 (12.24)	5 (10.20)	4 (8.20)		
BMI, kg/m^2^					
	23.26 ± 2.87	22.40 ± 3.17	21.84 ± 3.07	2.730	0.069^a^
Disease duration, years					
				1.608	0.807^c^
<5	11 (22.45)	12 (24.50)	15 (30.60)		
5–10	18 (36.73)	15 (30.60)	17 (34.70)		
>10	20 (40.82)	22 (44.90)	17 (34.70)		
GOLD levels					
					0.852^b^
GOLD II	21 (42.86)	19 (38.80)	23 (47.00)		
GOLD III	24 (48.98)	25 (51.00)	20 (40.80)		
GOLD IV	4 (8.16)	5 (10.20)	6 (12.20)		
Marital status					
				1.195	0.550^c^
Never married or divorced or widowed	8 (16.33)	10 (20.40)	6 (12.20)		
Married	41 (83.67)	39 (79.60)	43 (87.80)		
Education					
					0.957^b^
Primary school	9 (18.37)	13 (26.53)	10 (20.41)		
Middle school	26 (53.06)	25 (51.02)	28 (57.14)		
High school	3 (6.12)	4 (8.16)	2 (4.08)		
Some college or technical school	9 (18.37)	6 (12.24)	7 (14.29)		
College or postgraduate	2 (4.08)	1 (2.04)	2 (4.08)		
Monthly income, RMB, ¥					
				1.131	0.568^c^
<5,000	34 (69.39)	29 (59.20)	32 (65.30)		
≥5,000	15 (30.61)	20 (40.80)	17 (34.70)		
Smoking status					
				1.769	0.778^c^
Current smoker	17 (34.69)	18 (36.70)	14 (28.60)		
Former smoker	27 (55.10)	23 (46.90)	28 (57.10)		
Never smoked	5 (10.20)	8 (16.30)	7 (14.30)		
Hospitalizations in the past year					
					0.944^b^
None	19 (38.78)	21 (42.90)	21 (42.90)		
One time	26 (53.06)	23 (46.90)	25 (51.00)		
Two or more times	4 (8.16)	5 (10.20)	3 (6.10)		
CAT					
	21.63 ± 4.52	21.82 ± 3.58	21.02 ± 4.77	0.456	0.635^a^
Ex-SRES					
	68.94 ± 21.09	71.80 ± 21.35	70.59 ± 19.58	0.236	0.790^a^
Positive affect					
	20.33 ± 4.53	20.67 ± 4.38	20.39 ± 4.23	0.088	0.916^a^
mMRC					
	3(2,3)	3(2,3)	3(2,3.5)	1.075	0.584^d^
BREQ-2					
	4.00 ± 5.91	4.20 ± 5.91	5.04 ± 6.08	0.419	0.659^a^

^a^One-way analysis of variance; ^b^Fisher’s exact test; ^c^Chi-square test; ^d^Kruskal-Wallis H test.

### 3.3 Main evaluation indicators

**Figure 3:**
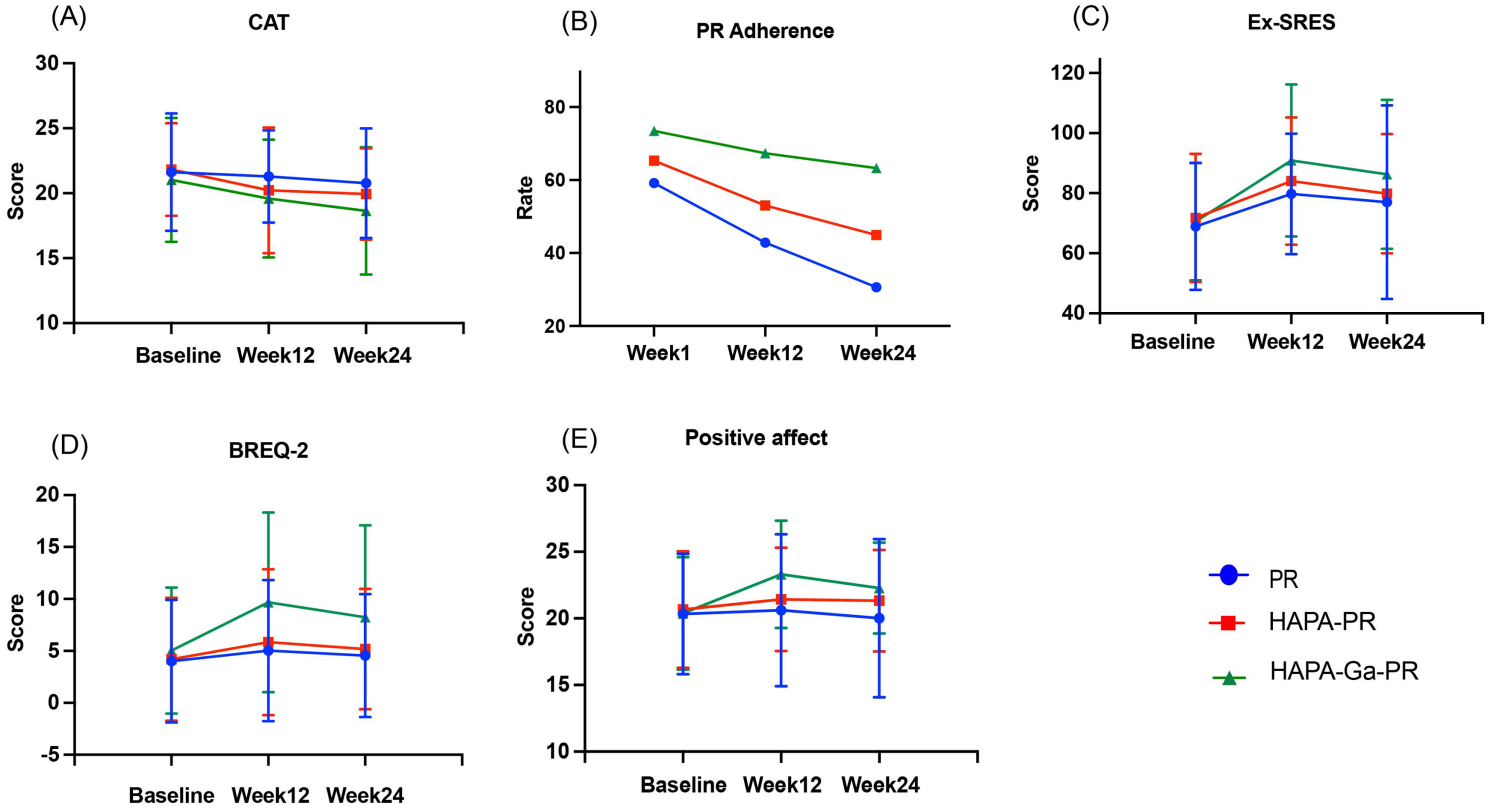
The changes of evaluation indicators over time.

Compared with adherence at week 1, the proportion of patients with high adherence in the PR group was statistically significant at week 12 (59.18%vs42.86%, *p* = 0.031), but not in the HAPA-PR and HAPA-Ga-PR groups (HAPA-PR: 65.31%vs53.06%, *p* > 0.05; HAPA-Ga-PR: 73.47%vs67.35%, *p* > 0.05). Compared with adherence at week 1, the proportion of patients with high adherence in the PR and HAPA-PR groups was statistically significantly lower at week 24 (PR: 59.18%vs30.61%, *p* = 0.001; HAPA-PR: 65.31%vs44.90%, *p* = 0.013), but did not occur in HAPA-Ga-PR group (73.47%vs63.27%, *p* > 0.05). At weeks 12 and 24, the proportion of patients with high adherence in the HAPA-Ga-PR group was significantly higher than that in the PR group (67.35%vs42.86%, *p* = 0.015; 63.27%vs30.61%, *p* = 0.001). Compared with baseline, CAT scores in HAPA-PR group and HAPA-Ga-PR group were significantly reduced at week 24 (*p* = 0.020, *p* = 0.032), but CAT scores in PR group had no significant difference (*p* > 0.05). At week 24, The CAT score of HAPA-Ga-PR group decreased significantly more than that of PR group (*p* = 0.039), and the proportion of patients in the HAPA-Ga-PR group whose CAT score change reached the minimum clinically important difference value was significantly higher than that in the PR group (69.39% vs. 26.53%, *p* < 0.001). The trend of CAT scores and the proportion of patients with high adherence over time is shown in Figures 3A,B. The main effect of group was statistically significant in CAT score (*p* = 0.041), but the main effect of time was not statistically significant (*p* > 0.05). There was statistical significance in the time main effect of adherence (*p* = 0.002), and there was no statistical significance in the group main effect (*p* > 0.05). The group × time interaction effect of the two main evaluation indicators had no statistical significance (*p* > 0.05).

### 3.4 Secondary evaluation indicators

Compared with baseline, exercise self-efficacy in the PR, HAPA-PR, and HAPA-Ga-PR groups improved significantly at week 12 (PR: MD = 10.84; 95% CI, 0.13 to 21.54; *p* = 0.046; HAPA-PR: MD = 12.25; 95% CI, 1.44 to 23.05; *p* = 0.022; HAPA-Ga-PR: MD = 20.29; 95% CI, 8.88 to 31.69; *p* < 0.001). Compared with baseline, only in the HAPA-Ga-PR group, exercise self-efficacy was still significantly improved at week 24 (MD = 15.71; 95% CI, 2.77 to 28.66; *p* = 0.013). At week 12, the exercise self-efficacy of patients in HAPA-Ga-PR group was higher than that in PR group, and the difference was statistically significant (MD = 11.10; 95% CI, 0.43 to 21.78; *p* = 0. 039). Figure 3C shows the trend of exercise self-efficacy over time. Compared with baseline, at week 12, HAPA-Ga-PR group significantly increased exercise motivation (MD = 4.63; 95% CI, 0.54 to 8.73; *p* = 0.022), but there was no statistical difference in exercise motivation between the PR group and the HAPA-PR group (*p* > 0.05). At the week 12, the exercise motivation of HAPA-Ga-PR group was significantly higher than that of PR group and HAPA-PR group (HAPA-Ga-PR vs. PR: MD = 4.63; 95% CI, 1.04 to 8.22; *p* = 0.008; HAPA-Ga-PR vs. HAPA-PR: MD = 3.67; 95% CI, 0.08 to 7.27; *p* = 0.044). At week 24, the exercise motivation of HAPA-Ga-PR group was still significantly higher than that of PR group (MD = 3.69; 95% CI, 0.34 to 7.04; *p* = 0.027). Figure 3D shows the trend of exercise motivation over time. Compared with baseline, HAPA-Ga-PR group showed a significant improvement in positive affect at week 12 (MD = 2.92; 95% CI, 1.11 to 4.73; *p* = 0.001), but there was no statistical difference in positive affect between the PR group and the HAPA-PR group (*p* > 0.05). At the week 12, positive affect in HAPA-Ga-PR group were significantly higher than those in PR and HAPA-PR group (HAPA-Ga-PR vs. PR: MD = 2.69; 95% CI, 0.85 to 4.54; *p* = 0.004; HAPA-Ga-PR vs. HAPA-PR: MD = 1.88; 95% CI, 0.03 to 3.72; *p* = 0.046). At the week 24, HAPA-Ga-PR group was still significantly higher than PR group (MD = 2.24; 95% CI, 0.44 to 4.05; *p* = 0.015). Figure 3E shows the trend of positive affect over time. Compared with baseline, at week 12, there were significant differences in mMRC scores only in the HAPA-Ga-PR group (*p* < 0.001), and at week 24, there were significant differences in all three groups (PR: *p* = 0.002; HAPA-PR: *p* = 0.020; HAPA-Ga-PR: *p* < 0.001), however, there was no statistically significant difference in mMRC scores among the three groups at baseline, weeks 12 and 24 (*p* > 0.05). The data of between-group differences are shown in Table 2, and the data of within-group differences are shown in Table 3. There was no statistically significant group × time interaction effect on the above indicators (*p* > 0.05).

**Table 2:** Between-group differences of outcomes.

Time	PR (*n* = 49)	HAPA-PR (*n* = 49)	HAPA-Ga-PR (*n* = 49)	Between-group differences					
				HAPA-PR vs PR		HAPA-Ga-PR vs PR		HAPA-Ga-PR vs HAPA-PR	
	*x̄ ± s /* M (P_25_, P_75_) / n (%)	*x̄ ± s* / M (P_25_, P_75_) / n (%)	*x̄ ± s* / M (P_25_, P_75_) / n (%)	MD (95%CI)/Z	*p*	MD (95%CI)/Z	*p*	MD (95%CI)/Z	*p*
CAT									
BL	21.63 ± 4.52	21.82 ± 3.58	21.02 ± 4.77	0.18 (−1.88–2.25)	0.976	−0.61 (−2.68–1.45)	0.763	−0.80 (−2.86–1.27)	0.634
Week 12	21.29 ± 3.56	20.22 ± 4.84	19.59 ± 4.54	−1.06 (−3.14–1.02)	0.451	−1.69 (−3.77–0.39)	0.135	−0.63 (−2.71–1.45)	0.752
Week 24	20.78 ± 4.22	19.94 ± 3.51	18.65 ± 4.91	−0.84 (−2.87–1.20)	0.595	−2.12 (−4.16--0.09)	0.039	−1.29 (−3.32–0.75)	0.296
PR Adherence									
Week 1	29 (59.18)	32 (65.31)	36 (73.47)	0.391	0.532	2.239	0.135	0.769	0.381
Week 12	21 (42.86)	26 (53.06)	33 (67.35)	1.022	0.312	5.939	0.015	2.087	0.149
Week 24	15 (30.61)	22 (44.90)	31 (63.27)	2.128	0.145	10.488	0.001	3.328	0.068
Ex-SRES									
BL	68.94 ± 21.09	71.80 ± 21.35	70.59 ± 19.58	2.86 (−7.04–12.75)	0.773	1.65 (−8.24–11.55)	0.917	−1.20 (−11.10–8.69)	0.955
Week 12	79.78 ± 20.06	84.04 ± 21.20	90.88 ± 25.32	4.27 (−6.41–14.94)	0.612	11.10 (0.43–21.78)	0.039	6.84 (−3.84–17.51)	0.286
Week 24	77.04 ± 32.24	79.86 ± 19.87	86.31 ± 24.83	2.82 (−9.69–15.33)	0.855	9.27 (−3.24–21.77)	0.189	6.45 (−6.06–18.96)	0.443
BREQ-2									
BL	4.00 ± 5.91	4.20 ± 5.91	5.04 ± 6.08	0.20 (−2.65–3.06)	0.984	1.04 (−1.81–3.89)	0.664	0.84 (−2.02–3.69)	0.767
Week 12	5.04 ± 6.80	6.00 ± 6.92	9.67 ± 8.66	0.96 (−2.63–4.55)	0.803	4.63 (1.04–8.22)	0.008	3.67 (0.08–7.27)	0.044
Week 24	4.55 ± 5.92	5.18 ± 5.80	8.24 ± 8.86	0.63 (−2.72–3.98)	0.896	3.69 (0.34–7.04)	0.027	3.06 (−0.29–6.41)	0.081
Positive affect									
BL	20.33 ± 4.53	20.67 ± 4.38	20.39 ± 4.23	0.34 (−1.40–2.10)	0.696	0.06 (−1.69–1.81)	0.945	−0.29 (−2.04–1.46)	0.747
Week 12	20.61 ± 5.71	21.43 ± 3.88	23.31 ± 4.02	0.82 (−1.03–2.66)	0.383	2.69 (0.85–4.54)	0.004	1.88 (0.03–3.72)	0.046
Week 24	20.02 ± 5.94	21.33 ± 3.80	22.27 ± 3.41	1.31 (−0.50–3.11)	0.155	2.24 (0.44–4.05)	0.015	0.94 (−0.87–2.75)	0.306
mMRC									
BL	3 (2, 3)	3 (2, 3)	3 (2, 3.5)	−0.691	0.489	0.315	0.753	1.014	0.311
Week 12	2 (2, 3)	2 (2, 3)	2 (2, 3)	−0.298	0.766	−0.569	0.569	−0.289	0.772
Week 24	2 (2, 3)	2 (2, 3)	2 (2, 3)	0.074	0.941	−0.282	0.778	−0.464	0.642

**Table 3:** Within-group differences of outcomes.

Time	Within-group differences					
	PR (*n* = 49)		HAPA-PR (*n* = 49)		HAPA-Ga-PR (*n* = 49)	
	MD (95%CI)/Z	*p*	MD (95%CI)/Z	*p*	MD (95%CI)/Z	*p*
CAT						
Week12 vs. BL	−0.35 (−2.24–1.55)	0.958	−1.59 (−3.59–0.40)	0.153	−1.43 (−3.06–0.20)	0.101
Week24 vs. BL	−0.86 (−2.72–1.01)	0.595	−1.88 (−3.52– −0.23)	0.02	−2.37 (−4.58– −0.16)	0.032
PR Adherence						
Week12 vs. Week1	4.500	0.031	2.083	0.146	0.235	0.629
Week24 vs. Week1	9.389	0.001	5.786	0.013	1.231	0.267
Ex-SRES						
Week12 vs. BL	10.84 (0.13–21.54)	0.046	12.25 (1.44–23.05)	0.022	20.29 (8.88–31.69)	<0.001
Week24 vs. BL	8.10 (−4.43–20.63)	0.310	8.06 (−3.43–19.55)	0.244	15.71 (2.77–28.66)	0.013
BREQ-2						
Week12 vs. BL	1.04 (−2.17–4.25)	0.811	1.80 (−1.57–5.17)	0.476	4.63 (0.54–8.73)	0.022
Week24 vs. BL	0.55 (−2.83–3.93)	0.970	0.98 (−1.33–3.29)	0.655	3.20 (−0.95–7.35)	0.175
Positive affect						
Week12 vs. BL	0.29 (−2.12–2.74)	0.989	0.76 (−1.11–2.62)	0.687	2.92 (1.11–4.73)	0.001
Week24 vs. BL	−0.31 (−3.01–2.39)	0.989	0.65 (−1.07–2.38)	0.729	1.88 (−0.18–3.94)	0.084
mMRC						
Week12 vs. BL	−1.890	0.059	−0.378	0.705	−3.742	<0.001
Week24 vs. BL	−3.153	0.002	−2.333	0.020	−4.583	<0.001

## 4 Discussion

The results of this study indicate that remote gamification PR based on HAPA theory is superior to remote PR based on self-efficacy in improving PR adherence, quality of life, exercise self-efficacy, exercise motivation and positive affect in COPD patients. Furthermore, remote gamification PR interventions based on HAPA theory were more effective than HAPA-based PR in enhancing motivation and positive affect. It is worth noting that remote gamification PR based on HAPA theory has long-term effects on the improvement of exercise self-efficacy and the maintenance of PR adherence.

In recent years, the combination of gamification and health intervention has attracted wide attention, which can significantly improve patient engagement and promote healthy behavior (37, 38). Behavioral intervention that combines gamification with theory is the current trend. In this context, this study combines gamification with the HAPA theory, introduces gamification elements, displays health data. And through cartoon-style animation, the degree of dyspnea of patients in daily scenes is presented to realize the perception and visualization of rehabilitation effects, so as to promote self-efficacy at various stages. This digital display method aims to realize the personalization, visualization and immersion of health data (39), so as to provide more vivid and intuitive feedback for the daily management of COPD patients, and enhance the self-efficacy of patients at different stages. This combination has the following advantages: Firstly, this study further expands the combination of gamification and HAPA theory. At present, some researchers have combined HAPA theory with gamification to construct intervention. In the study of Dadaczynski K et al. (40) researchers promoted patients’ intention to be physically active by developing goals and action plans based on travel tasks in different virtual cities. This study also promoted user self-efficacy through gamification of points, achievement badges, leaderboards and map progress displays. In the study of Orte S et al. (41) researchers helped users set dietary goals through the task progression of gamification, and promoted the maintenance of behaviors through gamification monitoring and system feedback. Although these studies have promoted healthy behavior in patients in different contexts, they have not fully explored how gamification can deepen self-efficacy as a key motivator in the HAPA framework, especially how it can effectively deepen and combine self-efficacy at multiple stages, such as behavior maintenance and behavior recovery. In this study, cartoon style avatars were used to simulate patients’ daily activity status to promote self-efficacy at various stages, including action coping self-efficacy, action maintenance self-efficacy and action recovery self-efficacy. Secondly, this approach effectively addresses the strategic challenge of applying HAPA theory to practical health interventions. In the intervention process of applying HAPA theory, there is a gap between intention and continuous action, which needs effective methods to bridge (42). Gamification enhances the dynamics in HAPA theory, especially self-efficacy, and HAPA provides a systematic and structured framework for ensuring that gamification elements lead to sustainable behavior change (43). This mutually supportive and integrated approach facilitates the application of HAPA theory while enhancing its applicability in digital health interventions. Future studies may consider applying this comprehensive intervention strategy to the management of other chronic diseases to further verify its applicability and effectiveness and provide more empirical evidence for digital health interventions.

The results of this study showed that by combining gamification with HAPA theory, not only had a positive impact on the behavioral level of COPD patients, but also significantly improved their positive affect and exercise motivation. This result can be explained from the perspective of affectal heuristic theory, which states that positive affect can enhance the rapidity and intuitiveness of individuals in decision-making (44, 45). In this study, gamification promoted positive affect by rewarding trophies and cartoon-style characters to visually see their progress. After experiencing this positive affect, patients were more willing to accept delayed gratification and sunk costs, which made them more willing to make healthy behavioral decisions, thus forming a virtuous circle. The two-way feedback loop of affect and behavior can help patients better maintain the continuity of healthy behaviors. In addition, the effect of positive affect on motivation is also worth exploring. According to the theory of affect motivation, which emphasizes the core role of affect in the formation and maintenance of motivation, affect not only reflect the inner state of an individual, but also affect the motivation of an individual, and positive affect can increase the willingness and persistence of an individual to pursue goals (46). In this study, gamification further enhances exercise motivation by stimulating patients’ positive emotions, which explains the potential mechanism of behavior change in COPD patients from a psychological perspective and also provides a reference for future digital medical interventions. The effectiveness of affect heuristic decision-making can help digital health platforms design more interactive and engaging content, further enhancing patient initiative and continuity in the healthcare management process.

In gamification interventions, the change of subsequent effects is a key issue (47). The results of this study showed that the initial effect of gamification was obvious, but it was difficult to maintain the effect, and adherence and motivation decreased during the follow-up period. Although gamification can rapidly enhance patients’ positive affect and promote healthy behaviors through incentives, tasks and feedback at the initial stage of intervention, the attenuation of subsequent effects becomes more and more obvious over time (48, 49). The reasons for the attenuation of the subsequent effects may be as follows: Firstly, with repeated use of gamification elements, patients may experience affect fatigue. This corresponds to the theory of affect heuristic decision making. Although positive affect can promote decision making and behavior in the short term, when external incentives are repeated, patients may gradually become immune to these incentives and their affect responses will weaken (50). This phenomenon of affect fatigue is usually manifested as progressive weakening of the patient’s response to the reward mechanism, potentially leading to boredom, which affects the maintenance of behavior. In addition, the “novelty effect” is also one of the important factors leading to the attenuation of subsequent effects. The initial interaction with game elements usually weakens over time, and the fading of this “novelty effect” may undermine the long-term effect (51). Therefore, in order to solve the problem of “novelty effect,” artificial intelligence combined with big data is an effective way to solve this problem. In the future, artificial intelligence or big data can be used to predict behavioral patterns and affect states of patients through data analysis, adjust game content and challenge difficulty in real time, and timely push personalized incentives or feedback when user engagement declines (52). Meanwhile, future iterations should take into account constantly changing challenges, regular content updates, and personalized difficulty adjustments to maintain engagement beyond initial interest. For example, new game elements, tasks or reward mechanisms can be introduced regularly to maintain the continuous attention and participation of patients. In summary, while gamification significantly promotes healthy behaviors and positive affect in the short term, sustained long-term effects require dynamic adjustments and personalized design to enhance continued user engagement. Future research should continue to explore innovative designs to improve the durability and effectiveness of gamification interventions.

By combining HAPA theory with gamification, this study transforms theoretical concepts into interactive experiences that adapt to the development of modern digital healthcare. This intervention model not only improves patient health outcomes and health behaviors, but also enhances the overall efficiency and effectiveness of digital care services. First of all, nurses can monitor patients’ rehabilitation progress through real-time data and adjust intervention strategies based on feedback. This dynamic response mechanism improves the routinization of nursing services and ensures the continuous improvement of patient participation. Secondly, gamification intervention significantly improved patients’ self-efficacy, affect engagement and rehabilitation motivation through online rewards and visual display of rehabilitation effects. This way not only enhances the sense of service experience, but also promotes the interaction between nurses and patients, achieving the goal of a win-win situation between nurses and patients. While optimizing service quality, this innovative model also effectively improves the long-term maintenance efficiency of rehabilitation behavior. To sum up, gamification intervention based on HAPA theory is not only a supplement to the traditional nursing model, but also an innovation in nursing services. By improving the rehabilitation experience and results of patients, it shows the great potential of digital medicine in the modern medical system, and promotes the synchronous improvement of medical service quality and efficiency. Future research can delve deeply into factors related to practical applications, including cost-effectiveness, resource requirements, and integration with existing healthcare workflows, to ensure the feasibility and sustainability of intervention measures in the actual medical environment.

This study has limitations. Firstly, although this study has potential application value in the rehabilitation of chronic diseases, the intervention measures of this study were specifically designed for patients with COPD. Other chronic diseases have significant differences from COPD in terms of pathology, symptoms and needs. The intervention content and visual presentation need to be further adjusted and optimized. Future studies should focus on different chronic diseases, formulate and evaluate targeted intervention plans. Secondly, adherence data mainly relies on self-reports. Future research can combine digital indicators such as application usage patterns or exercise completion timestamps to enhance the objectivity of adherence data. Meanwhile, the research lacks objective functional performance measurement standards, such as the 6-min walk test, etc. Such standardized evaluations should be included in the future to measure the intervention effect more comprehensively. Thirdly, the current research’s exploration of how patient characteristics affect intervention outcomes is limited. In the future, more detailed analyses can be conducted to examine the impact of factors such as GOLD staging, comorbidities profile, and baseline digital literacy on the intervention response, thereby clarifying which patients can benefit the most from this method. Fourthly, this study observed that the adherence of patients with pulmonary rehabilitation decreased at week 12 and 24, indicating the need to extend the follow-up time. Future studies could extend the follow-up period to more accurately assess long-term adherence and the persistence of intervention effects.

## 5 Conclusion

The results of this study highlight the importance of the remote PR model, especially in supporting personalized and digital management in older patients with COPD. The combination of gamification and HAPA theory not only has a positive impact on the behavioral level, but also significantly improves the positive affect and motivation of COPD patients, forming a virtuous cycle of affect and behaviors. By combining HAPA with modern digital technologies, this study opens up new paths for long-term management of chronic diseases and provides a valuable reference for personalized and digital interventions for COPD patients in the future.

## Data Availability

The raw data supporting the conclusions of this article will be made available by the authors, without undue reservation.

## Glossary

PR: pulmonary rehabilitation
COPD: chronic obstructive pulmonary disease
HAPA: health action process theory
CAT: COPD assessment test
HAPA-PR: pulmonary rehabilitation based on health action process approach theory
HAPA-Ga-PR: pulmonary rehabilitation based on health action process approach theory combined with gamification
PeR: Pulmonary Internet Explorer Rehabilitation
PDA: patient decision aid
FEV1: forced expiratory volume in 1 second
FVC: forced vital capacity
Ex-SRES: exercise self-regulatory efficacy scale
BREQ-2: behavioral regulation in exercise questionnaire-2
mMRC: modified Medical Research Council Dyspnea scale
ANOVA: analysis of variance
MD: Median Difference
CI: confidence interval
Z: Z-score
M: median
P_25_: 25th percentile
P_75_: 75th percentile
