# Supplementary material for: Effectiveness of remote gamification pulmonary rehabilitation intervention based on the health action process approach theory in older adults with chronic obstructive pulmonary disease: a pilot randomized controlled trial
Source: Front Med (Lausanne). 2025 Jun 19;12:1576256. doi: 10.3389/fmed.2025.1576256 (PMC12221897; doi:10.3389/fmed.2025.1576256)
Supplement: Supplementary file 2 [file Data_Sheet_2.PDF]

Usability testing *2*

An overview of usability testing 2

Method 2

Participants 2

TASKS 3

PROCEDURE 3

DATA SCORING 3

RESULTS 4

Theoretical mapping table *4*

Usability testing

An overview of usability testing

The usability of the HAPA-Ga-PR-component (Version 1) was assessed in a home simulation laboratory, where its effectiveness, efficiency, and user satisfaction were the primary evaluation criteria. In addition, participant experiences were gathered through user interviews. A purposive sampling method was used to recruit elderly COPD patients who met the specified inclusion and exclusion criteria for the study. The component was employed for simulation and presentation tasks during the test. Results demonstrated that the toolkit excelled in terms of effectiveness, efficiency, and user satisfaction, with participants indicating a high likelihood of recommending it to others.

Method

In this study, a mixed method was used to test HAPA-Ga-PR components. After r participant recruitment, the research assistant instructed them to complete a predefined core task while observing their task completion and timing. Subsequently, participants completed questionnaires and participated in user interviews.

Participants

Table 1 Characteristics of the participants (n=20)

Measures	$\bar{x}\pm s$ / n%
**Age**	68.55±7.34
**Sex**	
Male	17 (85.00)
Female	3 (15.00)
**Education**	
Uneducated	0 (0.00)
Primary school	2 (10.00)
Junior high school	5 (25.00)
Technical secondary school	3 (15.00)
High school	2 (10.00)
Junior college	5 (25.00)
Undergraduate	3 (15.00)
Master’s degree or above	0 (0.00)
**Place of residence**	
Cities	8 (40.00)
Towns	10 (50.00)
Rural areas	2(10.00)
**Monthly income (RMB, ¥)**	
<5000	4 (20.00)
≥5000	16 (80.00)
**Type of residence**	

Three generations together	5 (25.00)
Two generations together	3 (15.00)
Intergeneration	2 (10.00)
Couple lives	9 (45.00)
Live alone	1 (5.00)

TASKS

The study developed four core tasks, informed by the researchers' understanding of the frequency with which users engage with these features in real-world scenarios, as well as the potential challenges they may face. The specifics of these tasks are outlined in Table 2.

Table 2 Core tasks design

Number	Name	Description
Task 1	Knowledge learning	Click on PDA to see all about pulmonary rehabilitation
Task 2	Situational experience	Choose "Do exercises" from the cartoon scene for recovery exercise.
Task 3	Rehabilitation report	Please report the number and duration of exercise this week
Task 4	Reward feedback	After completing a week of rehabilitation training, check the feedback rewards provided by the system.

PROCEDURE

After the completion of the core tasks, participants were asked to fill out the PSSUQ, NPS, and CES questionnaires. Additionally, user interviews were conducted to delve into their overall user experience.

DATA SCORING

The scoring details for the core tasks are as follows:

Task Completion Rate:

The study involved four core tasks, with task failure occurring if any task was not completed. Completion rate was calculated as a percentage. Before starting, participants received instructions on system navigation. Task failure was defined as: (1) abandonment of the task, (2) needing assistance from the research assistant, or (3) performing actions outside the task scope. For example, if 10 out of 20 participants completed all tasks, the completion rate would be 50%.

Task Completion Time:

Participants completed tasks at their own pace without a time limit. Task duration was measured from the "start" signal until completion. Only successful tasks were included in the average calculation.

Questionnaires:

Participants completed questionnaires independently, with assistance available if needed. The PSSUQ assessed system utility, information quality, interface quality, and satisfaction. The CES was scored on a 1-7 scale, with the average calculated by dividing the total score by the number of participants. The NPS was scored on a 0-10 scale, with the result calculated as (Promoters - Detractors) / total sample size.

The following provides details of the scoring for the core tasks.

RESULTS

The usability test results, calculated based on the scoring criteria outlined above, are presented in Table 3.

Table 3 Results of usability test

Issues	n% / $\bar{x}$±s / n
Task completion rate	100
Task completion time	
Task 1 / s	13.35±3.70
Task 2 / s	12.80±4.71
Task 3 / s	17.64±4.31
Task 4 / s	14.32±3.55
**PSSUQ**	
System usefulness / 42	28.75
Information quality / 42	33.55
Interface quality / 49	35.45
Overall evaluation / 7	5.05
NPS	4.50
CES	3.05

Theoretical mapping table

Theory			Strategy	Intervention content	Module
HAPA	Behavioral intent stage	Risk awareness, Behavioral belief	Using the existing patient decision aid (PDA) to help patients improve their	● Initial communication to establish a trusting relationship and an initial understanding of the patient's health status and needs.<br>● Personal virtual	My choice and decision

			risk awareness and clarify their behavior beliefs; Through the use of immersive gamification elements in gamification elements, patients can enhance their sense of immersion to help them improve their risk awareness and clarify their behavioral beliefs	cartoon image selection: When operating on the interface, patients first enter the "My choice" module and choose the most suitable virtual cartoon image (divided into two genders, male and female, each gender has four characters) to enhance the immersion of patients.<br>● Import PDA based on the plot of virtual cartoon characters to assist patients in pulmonary rehabilitation decisions: "You feel chest tightness and asthma, and then go to the hospital to be diagnosed with COPD, is recommended for pulmonary rehabilitation, in the face of this unfamiliar vocabulary, you can first understand, and then make a decision..."<br>● Provide a pulmonary rehabilitation PDA (via wechat) : including the concept and process of SDM, the use of PDA, value clarification training, COPD disease knowledge, rehabilitation options comparison, evidence-based evidence presentation, pulmonary rehabilitation precautions, value clarification training, and a list of questions. The PDA aims to raise patients' risk awareness about the severity and personal risks of COPD, and to convince	

				patients of the positive outcomes of pulmonary rehabilitation training through clear behavioral beliefs.<br>● PDA Training course (via wechat, divided into 3 sessions, 10 minutes each)<br>● Value clarification training to help patients understand the personal meaning of different rehabilitation options. Through the use of question prompt lists, patients are guided to think about and clarify their values and preferences, further clarifying their behavioral beliefs and motivating them to adopt positive health behaviors.	
		Behavioral intention	Make goals that best suit your own value preferences and clarify the patient's behavioral intentions	● Based on shared decision making and value clarification, list rehabilitation goals and determine the patient's behavioral intent. Recovery goals are divided into three categories: disease - and symptom-specific goals, functional goals, and basic goals.<br>● Target ranking and visual display based on evidence-based evidence: the listed rehabilitation goals are ranked according to whether they can be achieved, when they can be achieved and how important they are, and the feasibility and importance of each goal are evaluated using	My goal

				evidence-based evidence to ensure that the goal setting is scientific and reasonable.	
	Planning phase	Self-efficacy of action	According to the patient's own condition and sports preference, a personalized rehabilitation program is developed to promote the patient's self-efficacy in action	● Provide detailed educational materials and video tutorials. According to the patient's condition and exercise preference, a personalized pulmonary rehabilitation program is developed, including exercise training, respiratory adjustment, medication guidance and diet guidance, to help elderly COPD patients improve the rehabilitation effect and quality of life:<br>● Develop a personalized exercise plan: Patients make rehabilitation plans based on their exercise preferences in daily life, including rest time, time arrangement, exercise habits, exercise environment, exercise time period and the duration of each exercise.<br>● In the interface, you can select cartoon sports scenes (including 9 virtual animation scenes based on St. George's questionnaire), basic scenes (breathing exercises), daily scenes (walking, bathing, going up and down stairs, doing housework) and advanced scenes (shopping, doing exercises, running, going out for entertainment) that meet your preferences;	My plan

				● Identify and deal with obstacles to rehabilitation: While developing an action plan, help patients identify obstacles that may affect the implementation of rehabilitation programs, such as time conflicts, physical deficiencies, environmental constraints, etc. Develop solutions for each obstacle.		
	Execution phase	Actions maintain self-efficacy	Through immersion related game elements to enhance patients' immersion experience, promote the maintenance of self-efficacy; Enhancing social support and promoting maintenance of self-efficacy through socially relevant play elements; Through animated videos based on the daily breathing difficulties of virtual cartoon characters,	● Lung rehabilitation in the virtual exercise scene: The system appears the corresponding sports cartoon scene according to the rehabilitation exercise plan formulated by the patient, and the patient can perform lung rehabilitation exercise according to the movement guidance on the interface, and make time records.<br>● The system gives feedback and rewards according to the patient's exercise situation: at the end of each week, the system will give corresponding rewards according to the patient's exercise compliance. Compliance ≥75% to obtain gold trophy, compliance < 75% to obtain silver trophy. In addition, other trophies can be obtained, such as the patient who has been	Multi-functional exercise recreation area	Virtual exercise scene<br>My trophy<br>Anonymous leaderboard<br>Rehabilitation effect perception visualization<br>Circle of friends

			patients can perceive the rehabilitation effect and promote the maintenance of self-efficacy	exercising for three weeks in accordance with high compliance, can be awarded the "Persistence Talent Award".<br>● Anonymous leaderboard: An anonymous leaderboard is also added to the interface, and patients can check the number of days of exercise and the number of MEDALS they have won in an anonymous form;<br>● "Circle of Friends" communication plate: Patients can leave free messages in this communication module to discuss their own rehabilitation experience or share knowledge, mood, etc.;<br>● Patients complete self-report after home training every week: report whether they have completed the amount of exercise this week, and report the type and frequency of exercise.<br>● Visual perception of rehabilitation effects: Patients can perceive rehabilitation effects through animated videos based on daily breathing difficulties of virtual cartoon characters. Every 4 weeks, according to the current degree of dyspnea selected by the		

				patient, the system pushes an animated video to show the degree of dyspnea of the patient. The animated video library includes 5 scenes of mMRC.		
		Action restores self-efficacy	Verbal cues and positive and negative feedback of achievement-related game elements were used to promote patients' action recovery	● In addition to regular communication between nurses and patients every 4 weeks, when patients do not clock in for more than 2 weeks, the system reminds patients to carry out rehabilitation exercise, and allows patients to choose the obstacle factors (environment, self, etc.) that affect the implementation of rehabilitation program and the solution to deal with these obstacle factors.<br>● When patients do not clock in for more than 2 weeks, the system will remind: "Because you did not insist on exercise, xx% of people have surpassed you in the ranking!" "	System background	
